# Gut Microbes in Gynecologic Cancers: Causes or Biomarkers and Therapeutic Potential

**DOI:** 10.3389/fonc.2022.902695

**Published:** 2022-07-13

**Authors:** Mengzhen Han, Na Wang, Wenjie Han, Meng Ban, Tao Sun, Junnan Xu

**Affiliations:** ^1^ Department of Breast Medicine, Cancer Hospital of China Medical University, Liaoning Cancer Hospital, Shenyang, China; ^2^ Department of Pharmacology, Cancer Hospital of China Medical University, Liaoning Cancer Hospital, Shenyang, China; ^3^ Department of Bioinformatics, Liaoning Microhealth Biotechnology Co., Ltd, Shenyang, China; ^4^ Department of Breast Medicine, Key Laboratory of Liaoning Breast Cancer Research, Shenyang, China

**Keywords:** gut microbe, cervical cancer, ovarian cancer, endometrial cancer, probiotic, postmenopausal status

## Abstract

The human intestine is home to a variety of microorganisms. In healthy populations, the intestinal flora shares a degree of similarity and stability, and they have a role in the metabolism, immunological response, and physiological function of key organs. With the rapid advent of high-throughput sequencing in recent years, several researchers have found that dysbiosis of the human gut microflora potentially cause physical problems and gynecological malignancies among postmenopausal women. Besides, dysbiosis hinders tumor treatment. Nonetheless, the importance of maintaining homeostatic gut microbiota and the effective use of probiotics in the treatment of gynecological malignancies should not be disregarded. Moreover, intestinal flora regulation and the involvement of probiotics as well as associated biologically active substances in gynecological malignancies could be an adjuvant treatment modality related to surgery and chemoradiotherapy in the future. Herein, this article aims to review the potential relationship between gut microorganisms and postmenopausal status as well as gynecologic malignancies; then the relationship between gut microbes and early screening as well as therapeutic aspects. Also, we describe the role of probiotics in the prevention, treatment, and prognosis of gynecologic malignancies.

## Introduction

Cervical cancer, ovarian cancer, and endometrial cancer are the most common gynecological cancers. Based on the latest global cancer data, cervical cancer among women has the 4^th^ and 6^th^ highest incidence and mortality rates, respectively, whereas ovarian cancer has the 5^th^ highest mortality rate, topping the list of gynecological malignant tumors (1). Moreover, as the human living standards improve, obesity (2), endocrine disorders, environmental pollution may also increase the incidence and mortality of gynecological cancers. However, there is no clear conclusion on the causes and mechanisms of gynecologic malignancies. Thus, a discussion on the importance of intestinal flora to gynecologic malignancies and the potential role of probiotics help scholars investigate gynecologic malignancies from a different angle. This article reviews the microbial and gynecological malignancy importance from the following aspects: First is the effect of changes in gut microbes (GM) on the development of gynecological malignancies. Second is the hypothesis of biomarkers of gynecologic malignancies. Lastly are the aspects related to the treatment of gynecologic malignancies, such as the relationship between microorganisms and the sensitivity of chemotherapeutic agents including platinum drugs, as well as the use of probiotics and flora transplantation in the treatment and prognosis of gynecologic malignancies. Here, our objective is to investigate the effect of changes in body flora on gynecological malignancies geared towards providing novel insights for early screening and subsequent treatment of gynecological malignancies.

## Intestinal Microecology

### GM

The human GM comprise numerous groups of microorganisms including bacteria, archaea, viruses, eukaryotes, and parasites. The microbial community in the human gut is extremely large containing up to 150 times more genes than the human genome (3).The majority of these microbes are bacteria interacting with the human intestinal tract in the long-term evolution process; they primarily include *Bacteroides*, *Clostridium*, *Bifidobacterium*, and *Lactobacillus*. Pathogenic flora is a small minority. Disruption of commensal flora or invasion of pathogenic bacteria including *Salmonella* (4), affects the homeostasis of he intestinal microenvironment, resulting in abdominal pain, diarrhea, and other diseases. Studies reveal that patients with gynecologic malignancies display changes in intestinal microflora before the onset and during treatment (5, 6), thereby suggesting a correlation between GM and gynecologic malignancies.

### GM and Cancer

**Figure 1:**
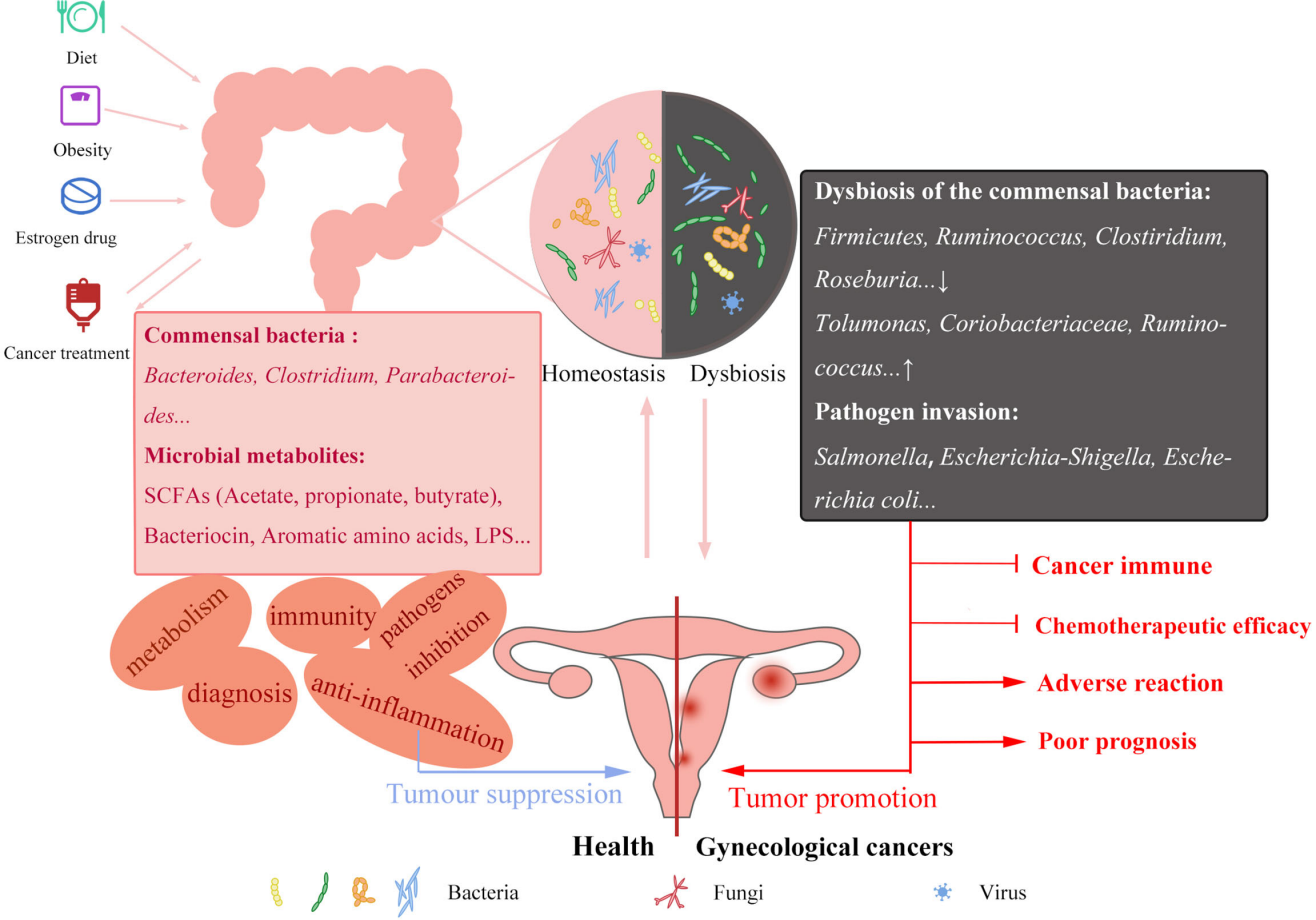
Factors affecting GM and its potential mechanisms of GM in gynecological cancers. Unhealthy diet, obesity, estrogen drug abuse and cancer treatment can affect the homeostasis of GM, commensal bacteria decreased and pathogenic bacteria increased. Interaction between GM and gynecological cancers. GM stabilization can inhibit the occurrence of cancer in immunity and preventing pathogen invasion. The reduced protective effect of the maladjusted GM will also play an adverse role in cancer immunity, chemotherapy efficacy and prognosis. In-depth study of GM changes in gynecological cancer patients can be applied to the screening, improve the treatment of gynecological cancers and improve the poor prognosis.

GM includes microorganisms residing in the human intestine; these microbes are living interdependently with the human body, and therefore their homeostasis plays a significant role in human health. GM is involved in the digestion and absorption of the body’s intake and modulates the development and treatment of diabetes (7), cardiovascular disease (8), osteoporosis (9), Alzheimer’s disease (10, 11), autism (12), depression (13), as well as cancer. Healthy and stable intestinal flora inhibits the development of cancer, whereas dysregulated intestinal flora has a limited protective effect on the body and could promote cancer development (**Figure 1**). Healthy individuals and colorectal cancer patients have significantly distinct gut flora structures. In contrast with healthy individuals, colorectal cancer patients have more abundance of *Bacteroides fragilis*, *Enterococcus*, *Escherichia/Shigella*, *Klebsiella*, *Streptococcus*, and *Peptostreptococcus*. Enrichment of opportunistic pathogens potentially promotes the occurrence of colorectal cancer by causing related inflammation. Besides, the decrease of butyric acid-producing bacteria is common among colorectal cancer patients. Butyric acid plays an important role in body immunity and inhibition of tumors *in vivo*. This means that an imbalance in GM diminishes the original protective effect and promotes the development of colorectal cancer (14). Also, GM may affect the efficacy of drugs during cancer treatment by targeting the immune response and disrupting the chemotherapeutic drug sensitivity (15, 16). At present, immunotherapy for cancer is a major hot topic in cancer treatment. As immunotherapies including CAR-T cells continue to mature, scholars are gradually shifting their focus to the impact of microbiota on cancer immunotherapy. One study found that CTLA-4 specific antibody displayed no effect on the tumor of antibiotic-treated and germ-free mice, whereas the presence of specific bacteria, including *B. Fragilis* had an important effect on the efficacy of CTLA-4 blockade. This indicates that the existence and structure of intestinal flora mediate the efficacy of tumor immunotherapy, nevertheless, research on specific mechanisms is still ongoing (17). Recent studies suggest that short-chain fatty acids, including butyric acid produced by intestinal microbial fermentation potentially affect T cell metabolism and related gene expression levels, thereby improving the activity of anti-tumor T cells. Besides, inosine, produced by intestinal bacteria *B.Pseudolusgum* potentially plays an auxiliary role in improving the efficacy of ICB immunotherapy (18). Additionally, the radiotherapy process in cancer patients causes dysbiosis of GM (5), which in turn becomes more detrimental to cancer treatment. The use of antibiotics, probiotics, fecal microbiota transplants (FMT), and certain metabolites to artificially manage the gut flora could provide a strong adjunct to cancer treatment. Mechanistic aspects of the effect of GM on cancer development are also a focus of future research.

## Gynecologic Malignancies and GM

### Flora Changes and Diseases in Postmenopausal Women

Most gynecological malignancies are predominant in postmenopausal and older women; therefore, it is necessary to explore changes in the intestinal flora and related diseases among postmenopausal women. Lack of endogenous estrogen in women after menopause triggers a wide range of problems, such as increased incidence of cardiovascular disease (19), osteoporosis (20), obesity (21), diabetes (22), breast cancer (23), and other gynecologic cancers. An increase in intestinal permeability has been noted during the menopausal transition; besides, there is a relationship between increased intestinal permeability and the development of inflammation (24). Additionally, unlike premenopausal women, fecal samples from postmenopausal women showed changes in the flora linked to endocrine disorders and osteoporosis, including a decrease in *Firmicutes* and *Roseburia* spp. and an increase in *Bacteroidetes* and toluene-producing genus *Tolumonas* (25). In turn, hormone replacement therapy (26), used to combat these conditions potentially increases the incidence of estrogen-related cancers including breast and ovarian cancers (27). As such, it is necessary to analyze the effect of microorganisms on estrogen levels and innovate microbial-assisted therapy or reduce the side effects of hormone replacement therapy. Previous studies reported that non-ovarian systemic estrogen levels are directly related to the richness and alpha diversity of intestinal flora and could affect their levels *via* enterohepatic circulation (28). This leads to thinking whether it is possible to control the disease from the source to reduce the incidence of the disease by artificially intervening the GM of menopausal and postmenopausal women. In addition, vaginal dryness and tissue atrophy in postmenopausal women markedly affect the quality of life. Altered environmental status of the vagina potentially conducive to the invasion of pathogenic bacteria from the vagina resulting in vaginal dysbiosis and gynecological malignancies. In one experiment, the fecal microbes of ovary-intact fecund female mice was transplanted into ovariectomized ones with vaginal atrophy, and consequently, the atrophy symptoms significantly improved. This indicates an interaction between the ovarian and reproductive tract status as well as the GM (29). Moreover, the improvement of vaginal epithelial atrophy symptoms by FMT could exhibit an impact on the prevention of gynecological malignancies by improving vaginal epithelial atrophy-related protective effects. In summary, investigating changes of GM in postmenopausal women and their roles in disease development as well as treatment will yield unprecedented effects on the health and quality of life among postmenopausal women.

### GM in Cervical Cancer

Cervical cancer is one of the most prevalent gynecological malignant tumors that threatens the life and health of women. The current research on the relationship between GM and cervical cancer primarily focuses on the changes of GM and related intestinal diseases among cervical cancer patients post-radiotherapy. The majority of scholars share a similar view on gut microbial alterations in cervical cancer patients (6). However, there is controversy on the research of intestinal biomarkers and a lack of research on related biological mechanisms. Through 16S rRNA sequencing comparison, scholars noted differences in GM between cervical cancer patients and healthy individuals. Based on recent reports, phylum *Proteobacteria*, genus *Parabacteroides*, *Escherichia_Shigells* and *Roseburia* were predicted to be potential biomarkers in the identification of cervical cancer (30). However, another study soon after showed different results, with Kang and colleagues identifying *Prevotella*, *Peptostreptococcus*, *Finegolida*, *Ruminococcus*, *Clostridium*, *Pseudomonas*, and *Turibacter* as biomarkers for predicting early cervical cancer (31). The aforementioned studies provide a future direction and act as a catalyst for the future upgrading of early prediction and diagnostic modalities for cervical cancer. In addition, the researchers discovered a decrease in butyrate-producing bacteria *Ruminococcus* and *Clostridium* in the intestine of patients with early-stage cervical cancer (31). Short-chain fatty acids (SCFAs) such as butyric acid produced by microbial metabolism have good antitumor activity (32–34), affecting various beneficial processes such as immunity and apoptosis of cancer cells,thus suggesting a possible indirect link between GM and cancer development through the presence or absence of specific genera and altered numbers. Additional experiments are therefore required to validate these possibilities for interrelationship between GM and cervical carcinogenesis and their roles in screening and diagnosis.

### GM in Ovarian Cancer

Since abdominal discomfort is a hallmark symptom of ovarian cancer and gastrointestinal adverse effects are evident during treatment, it is meaningful to study the association between GM and ovarian cancer (35). Dysbiosis of the gut microbial environment is strongly associated with the development of ovarian cancer. GM disorder promotes tumor growth and causes an epithelial-mesenchymal transition in xenograft mice (36). Additionally, the imbalance of GM is associated with obesity (37) and estrogen level (15); obesity and estrogen imbalance are risk factors for the incidence of ovarian cancer (2, 38). This indicates that imbalanced GM could cause obesity and estrogen imbalance *via* an approach that triggers ovarian cancer. Besides, a non-obese individual should regulate their diet. A diet high in oil, fat, and salt is also undesirable. A study on animals subjected to a high-fat diet reported the diet may promote the development of ovarian cancer by disrupting the levels of inflammatory factors (39). Therefore, poor diet and health habits are crucial risk factors for the occurrence of cancer.

Studies on ovarian cancer treatment indicate that GM influences the sensitivity of the human body to platinum drugs. The advert effects of chemotherapy on GM, e.g. dysbiosis, are more prevalent in patients (16) and animal (40) models bearing ovarian cancers. In addition, compared to platinum-sensitive patients, platinum-resistant patients showed a more pronounced dysbiosis of GM, with a decrease in the health-related groups and an increase in the proportion of lactic acid-producing bacteria including *Coriobacteriaceae* and *Bifidobacterium* (16). In addition to reduced platinum sensitivity, animal studies have revealed that the destruction of GM contributes to the growth of ovarian malignancies and reduces mice survival (41). Also, it indicates that the integrity and stability of GM regulate the efficacy of chemotherapy drugs. GM metabolites such as bile acids can interact with host drug metabolizing enzymes to affect drug disposition, pharmacokinetics and pharmacodynamics (42). The discovery of potential features of GM potentially provides a novel idea for future early detection, treatment, and even prognosis of epithelial ovarian cancer. GM and their products may also contribute to the treatment of ovarian cancer. Zhao and colleagues isolated four strains of *Bacillus* from the stool of healthy individuals and cancer patients and observed that bacterial products inhibit proliferation of ovarian cancer cells, possibly by causing apoptosis (43). These bacterial products may be optimized as anticancer drugs in the future. Furthermore, one study using a mouse model discovered that anticancer drugs have a certain effect on GM, causing the transfer of Gram-positive bacteria to secondary lymphoid organs and producing an immune response (44). This indicates that the intestinal microbiota favorably affects the formation of anticancer immune response and the efficacy of chemotherapy.

Gut microbial changes after ovarian cancer treatment should also be noticed. Unlike pre-operative stool samples of ovarian cancer, the abundance of *Bacteroidetes* and *Firmicutes* was significantly lower in post-operative stool samples, whereas *Proteobacteria* abundance was significantly higher. Similar changes were noted in chemotherapy group (5). This implies that the treatment of ovarian cancer has a significant impact on intestinal flora and that there may be a potential correlation between intestinal flora and the clinical prognosis of ovarian cancer patients.

### GM in Endometrial Cancer

Endometrial cancer is a group of epithelial malignant tumors of the endometrium that occur most frequently in perimenopausal and postmenopausal women. The risk factors of endometrial cancer include obesity, diabetes, and hypertension (45–47). The incidence of endometrial cancer rates varies dramatically not only between premenopausal and postmenopausal women but also among countries with varying degrees of development. Its incidence is higher in Europe and North America than in developing countries (48). Researchers speculate that the development of endometrial cancer could be related to the adverse consequences of hormonal abnormalities and overweight due to the improvement in the quality of life and population diet. The disruption of digestion and absorption induced by dysbiosis of GM potentially causes obesity, and the occurrence of obesity could result in hypertension, diabetes, and hormonal disorders. This suggests a relationship between GM and endometrial cancer. Therefore, analyzing the changes of intestinal flora in obese people potentially provides novel insights for the in-depth study of endometrial cancer. In addition, the incidence of endometrial cancer is associated with menstruation, marriage and childbirth, smoking, drinking, and other factors. Dietary habits, marriage and love policies and customs of different countries, environmental pollution, and even ethnic differences potentially lead to different incidences of endometrial cancer. GM are able to regulate systemic estrogen levels in women. β-glucuronidases and β-glucuronides encoded by estrobolome such as (49) *Bifidobacterium*, *Clostridium*, and *Lactobacillus* are able to act in the intestine to regulate circulating estrogen levels (15, 50). They remove the glucuronides of conjugated estrogens excreted *via* bile to obtain free estrogen molecules. In addition, hydroxysteroid dehydrogenases are also widely present in the human gut and are involved in the partial reduction process of estrogen synthesis from cholesterol precursors (51). Urinary (52) and serum (53) estrogen levels were positively correlated with gut microbial diversity. GM also confer biological activity to foreign estrogen-like compounds (54, 55). For example, GM are able to catalyze the metabolism of daidzein to the form of equol or O-desmethylangolensin (56). Lignans beneficial effects are also dependent on the activity of gut microbial metabolites enterodiol and enterolactone (57). Therefore, in addition to the direct effects of female genetic differences and altered physical conditions as well as environmental estrogen exposure (58) on hormone levels, the dysbiosis of GM due to poor lifestyle, diet, and antibiotic abuse also indirectly affects estrogen levels, which in turn promotes endometrial carcinogenesis. A recent review on the potential relationship between intestinal flora, obesity, menopausal status, estrogen, and endometrial cancer also suggests that menopause and obesity could modulate the development of endometrial cancer by causing changes in estrogen due to intestinal flora imbalance (59). Obesity contributes to an increased risk and a worse prognosis for endometrial cancer. One study discovered that the endometrial epithelial cells of obese women revealed numerous methylation changes, and 54 overlapping regions of differential methylation with stage I endometrial cancer. This suggests that obesity promotes the development of endometrial cancer by influencing DNA methylation and causing dysregulation of related metabolic pathways (60). The effect of obesity on endometrial cancer has led to speculation on whether bariatric surgery can help ameliorate obesity by stabilizing the GM and estrogen levels and possibly even promote the treatment of endometrial cancer in the future. A related study reported that bariatric surgery causes changes in the GM with beneficial effects, but not in terms of hormones (61), and there was no direct relationship between bariatric surgery and the incidence of endometrial cancer. However, digging into the potential role of bariatric surgery in endometrial cancer could have unprecedented application in future cancer research.

## The Potential Role of Probiotics in the Adjuvant Treatment of Gynecological Malignancies

**Figure 2:**
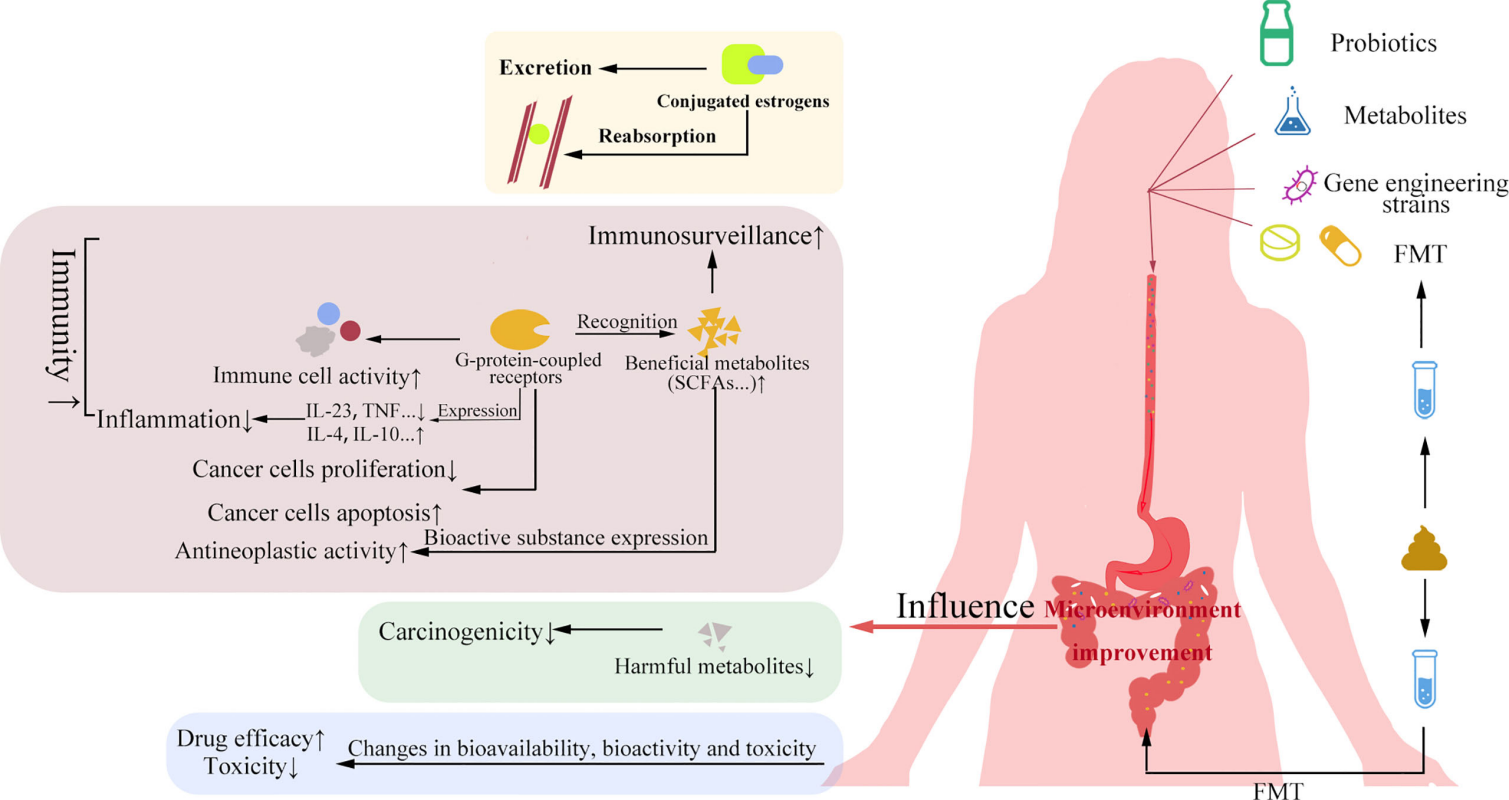
Significance of probiotics and FMT in gynecological cancers. Probiotics and their metabolites, genetic engineering strains and FMT can improve the gut microbial environment. The improvement of gut microbial environment can affect estrogen level, improve cancer immunity and chemotherapy efficacy, increase apoptosis of cancer cells, and reduce the rapid proliferation of cancer cells and drug side effects.

As the name suggests, probiotics are beneficial microorganisms to the body within a certain dose range. The management of probiotics can improve the dysbiosis of GM to certain extent, which in turn can have beneficial effects on gynecological cancers by affecting estrogen levels, cancer immunity, cancer cell proliferation and apoptosis, and drug resistance (**Figure 2**).

### Lactobacillus Rhamnosus

*Lactobacillus rhamnosus* is a star genus in the field of probiotics with wide range of applications. The potential role of *Lactobacillus rhamnosus* in cancer is widely divided into the following components. First, *Lactobacillus rhamnosus* modulates homeostasis of the intestinal flora and protects the mucosa and restores the intestinal barrier function by enhancing the expression of genes related to healthy intestinal permeability (62–64), whereas the homeostatic effect of intestinal flora on gynecological malignancies has been mentioned above. Secondly, regarding human self-protection, *Lactobacillus rhamnosus* potentially regulates immunity, causing apoptosis of cancer cells and anti-inflammatory effects by activating or inhibiting cytokine expression (65–67), and invasion of pathogens (68, 69). Eventually, *Lactobacillus rhamnosus* also protects the body from the toxicity and side effects of radiotherapy and other treatment methods (70, 71).

### Bacteria Isolated From Secretions or Excretions

Several studies have documented the anticancer activity of certain bacteria isolated from human and animal secretions or excretions. *Bifidobacterium adolescentis* SPM1005-A (72) isolated from human feces, *Lactobacillus plantarum* 5BL (73) isolated from vaginal secretions of adolescent females, *Lactobacillus gasseri* G10 and H15 (74) isolated from the vagina of healthy humans, three potential probiotic strains isolated from human milk *Lactobacillus casei* SR1, *Lactobacillus casei* SR2, and *Lactobacillus paracasei* SR4 (75), and *Enterococcus faecalis* por1 (76) isolated from porcine intestinal food, etc. have desired probiotic properties and may inhibit cancer development by targeting oncogenes, preventing cancer cell growth, inducing apoptosis or modulating immune response capacity.

### Metabolites of the Bacterium

Certain microbial-associated metabolites suppress gynecologic malignancy development. Kevin et al. found that the parasporin-2Aa1 isolated from *Bacillus thuringiensis* 4R2, activated by proteinase K, induces various human cancer cells from different tissues, including endometrial cancer cell of apoptosis, however, it does not affect normal cells (77). Due to its good properties, parasporin-2Aa1 can be used in the future to reduce the killing of normal cells in combination with chemotherapeutic drugs.

### Probiotic Blends and Probiotic Modifications

Probiotic blends and genetically engineered probiotics promote cancer treatment by targeting the immune system. Bermudez and colleagues showed that recombinant *Lactococci* engineered to express HPV-16 E7 antigen, administered intranasally, stimulates both the cell-mediated (which secretes IL-12 and IFN - γ) and humoral immune systems (which produces antibodies to E7) to prevent HPV-associated cervical cancer (78). Additionally, studies have shown that oral administration of probiotic mixture comprising specific *lactobacillus rhamnosus*, *Lactobacillus acidophilus*, and lactoferrin RCXTM regulate the innate and adaptive immune responses of the vagina and the whole body, weaken vaginopathy induced by *Gardasella vaginalis* (79), and prevent gynecological cancers resulting from inflammation.

### Role in Other Aspects Related to Gynecological Malignancies

Estrogen deficiency affects the health of women, nevertheless, improper estrogen supplementation could also cause diseases including breast and endometrial cancers. Therefore, the use of probiotics in combined administration with hormone-related drugs is a hot topic for future estrogen deficiency research. Bioavailable isoflavones with selective estrogen receptor affinity have potential in the prevention and treatment of osteoporosis due to estrogen deficiency, at the same time minimizing or eliminating its carcinogenic side effects (80). Also, probiotics modulate the prevention and management of cancer treatment side effects. One possible acute side effect of cervical cancer treatment through radiotherapy is radiation-induced diarrhea, and many experiments have demonstrated that supplementation with probiotics including *Lactobacillus lactis*, *Bifidobacterium animalis*, and *Lactobacillus acidophilus* minimize the incidence and severity of radiation-induced diarrhea in cervical cancer patients (81, 82). Most of the above studies are based on phenomena, and in-depth studies are needed to prove the accuracy of the conclusions. Research on the mechanism of action of probiotics in the treatment of gynecological cancers is still lacking and needs to be further explored.

## Prospect

Microorganisms are neither good nor bad by themselves; they are divided into beneficial and harmful bacteria for the host, resulting in a two-sided effect of microorganisms on the host. On one hand, dysbacteriosis and invasion of pathogenic bacteria promote carcinogenesis and are detrimental to the later treatment of gynecological malignancies. Nevertheless, the maintenance of flora homeostasis and the application of probiotics promote cancer inhibition. The effective use of gut microorganisms has a significant impact on the future of gynecologic malignancies. The above studies have promoted the advances in microbiological aspects of gynecological malignancies; however, there have been shortcomings including a small sample size and incomplete study of mechanisms. Therefore, future studies should increase the sample size, consider all relevant parameters influencing the results, and focus on investigating mechanisms and clinical effects for better application in practice.

In summary, the following recommendation will significantly prolong the survival time of patients with gynecological malignant tumors as well as improve the survival rate and quality of life: In-depth research on the relationship between the microbial flora *in vivo* and the generation as well as the development of gynecological malignant tumors at various stages; the use of microorganisms in predicting early stages of cancer; research on the effect of beneficial microorganisms and genetic engineering transformation; the use of probiotics; the transplantation of bacteria and other means to intervene and control the flora *in vivo* and the use of microorganisms to improve the sequelae after treatment of gynecological malignant tumor.

## Author Contributions

XJ and TS: Conceived and designed the review. MH and NW wrote the draft of paper. WH and MB drew the picture. All authors read and approved the final manuscript and submission of this manuscript.

## Funding

This work was supported by CSCO Project (Y-2019Genecast-019,JX), Liaoning Cancer Hospital Yangtse River Scholars Project (JX), Medical-Engineering Cross Research Fund between Liaoning Cancer Hospital & Dalian University of Technology (LD202022, TS) and LiaoNing Revitalization Talents Program (XLYC1907160, JX)

## Conflict of Interest

Author BM is employed by Liaoning Microhealth Biotechnology Co., Ltd.

The remaining authors declare that the research was conducted in the absence of any commercial or financial relationships that could be construed as a potential conflict of interest.

## Publisher’s Note

All claims expressed in this article are solely those of the authors and do not necessarily represent those of their affiliated organizations, or those of the publisher, the editors and the reviewers. Any product that may be evaluated in this article, or claim that may be made by its manufacturer, is not guaranteed or endorsed by the publisher.

## References

[1] Bray F Ferlay J Soerjomataram I Siegel RL Torre LA Jemal A. Global Cancer Statistics 2018: Globocan Estimates of Incidence and Mortality Worldwide for 36 Cancers in 185 Countries. CA Cancer J Clin (2018) 68(6):394–424. doi: 10.3322/caac.21492 30207593

[2] Avgerinos KI Spyrou N Mantzoros CS Dalamaga M. Obesity and Cancer Risk: Emerging Biological Mechanisms and Perspectives. Metabolism (2019) 92:121–35. doi: 10.1016/j.metabol.2018.11.001 30445141

[3] Qin J Li R Raes J Arumugam M Burgdorf KS Manichanh C. A Human Gut Microbial Gene Catalogue Established by Metagenomic Sequencing. Nature (2010) 464(7285):59–U70. doi: 10.1038/nature08821 20203603 PMC3779803

[4] Ferrari RG Rosario DKA Cunha-Neto A Mano SB Figueiredo EES Conte-Junior CA. Worldwide Epidemiology of Salmonella Serovars in Animal-Based Foods: A Meta-Analysis. Appl Environ Microbiol (2019) 85(14):e00591–19. doi: 10.1128/AEM.00591-19 31053586 PMC6606869

[5] Tong J Zhang X Fan Y Chen L Ma X Yu H. Changes of Intestinal Microbiota in Ovarian Cancer Patients Treated With Surgery and Chemotherapy. Cancer Manag Res (2020) 12:8125–35. doi: 10.2147/CMAR.S265205 PMC7494227 32982410

[6] Sims TT Colbert LE Zheng JL Medrano AYD Hoffman KL Ramondetta L. Gut Microbial Diversity and Genus-Level Differences Identified in Cervical Cancer Patients Versus Healthy Controls. Gynecol Oncol (2019) 155(2):237–44. doi: 10.1016/j.ygyno.2019.09.002 PMC6825899 31500892

[7] Sharma S Tripathi P. Gut Microbiome and Type 2 Diabetes: Where We Are and Where to Go? J Nutr Biochem (2019) 63:101–8. doi: 10.1016/j.jnutbio.2018.10.003 30366260

[8] Ahmad AF DG O'Gara F Caparros-Martin J Ward NC. The Gut Microbiome and Cardiovascular Disease: Current Knowledge and Clinical Potential. Am J Physiol Heart Circ Physiol (2019) 317(5):H923–38. doi: 10.1152/ajpheart.00376.2019 31469291

[9] Ding K Hua F Ding W. Gut Microbiome and Osteoporosis. Aging Dis (2020) 11(2):438–47. doi: 10.14336/AD.2019.0523 PMC7069453 32257552

[10] Zhuang ZQ Shen LL Li WW Fu X Zeng F Gui L. Gut Microbiota Is Altered in Patients With Alzheimer's Disease. J Alzheimers Dis (2018) 63(4):1337–46. doi: 10.3233/jad-180176 29758946

[11] Angelucci F Cechova K Amlerova J Hort J. Antibiotics, Gut Microbiota, and Alzheimer's Disease. J Neuroinflamm (2019) 16(1):108. doi: 10.1186/s12974-019-1494-4 PMC6530014 31118068

[12] Garcia-Gutierrez E Narbad A Rodriguez JM. Autism Spectrum Disorder Associated With Gut Microbiota at Immune, Metabolomic, and Neuroactive Level. Front Neurosci (2020) 14:578666. doi: 10.3389/fnins.2020.578666 33117122 PMC7578228

[13] Konjevod M Perkovic MN Saiz J Strac DS Barbas C Rojo D. Metabolomics Analysis of Microbiota-Gut-Brain Axis in Neurodegenerative and Psychiatric Diseases. J Pharm Biomed Anal (2021) 194:113681. doi: 10.1016/j.jpba.2020.113681 33279302

[14] Wang T Cai G Qiu Y Fei N Zhang M Pang X. Structural Segregation of Gut Microbiota Between Colorectal Cancer Patients and Healthy Volunteers. ISME J (2012) 6(2):320–9. doi: 10.1038/ismej.2011.109 PMC3260502 21850056

[15] Graham ME Herbert WG Song SD Raman HN Zhu JE Gonzalez PE. Gut and Vaginal Microbiomes on Steroids: Implications for Women's Health. Trends Endocrinol Metab (2021) 32(8):554–65. doi: 10.1016/j.tem.2021.04.014 PMC8282721 34049772

[16] D'Amico F Perrone AM Rampelli S Coluccelli S Barone M Ravegnini G. Gut Microbiota Dynamics During Chemotherapy in Epithelial Ovarian Cancer Patients Are Related to Therapeutic Outcome. Cancers (Basel) (2021) 13(16):3999. doi: 10.3390/cancers13163999 34439153 PMC8393652

[17] Vétizou M Pitt JM Daillère R Lepage P Waldschmitt N Flament C. Anticancer Immunotherapy by Ctla-4 Blockade Relies on the Gut Microbiota. Science (2015) 350(6264):1079–84. doi: 10.1126/science.aad1329 PMC4721659 26541610

[18] Luu M Visekruna A. Microbial Metabolites: Novel Therapeutic Tools for Boosting Cancer Therapies. Trends Cell Biol (2021) 31(11):873–5. doi: 10.1016/j.tcb.2021.08.005 34538658

[19] Zilberman JM. Menopause: Hypertension and Vascular Disease. Hipertension y riesgo Vasc (2018) 35(2):77–83. doi: 10.1016/j.hipert.2017.11.001 29396243

[20] Ikeda K Horie-Inoue K Inoue S. Functions of Estrogen and Estrogen Receptor Signaling on Skeletal Muscle. J Steroid Biochem Mol Biol (2019) 191:105375. doi: 10.1016/j.jsbmb.2019.105375 31067490

[21] Ko SH Kim HS. Menopause-Associated Lipid Metabolic Disorders and Foods Beneficial for Postmenopausal Women. Nutrients (2020) 12(1):202. doi: 10.3390/nu12010202 PMC7019719 31941004

[22] Mauvais-Jarvis F Clegg DJ Hevener AL. The Role of Estrogens in Control of Energy Balance and Glucose Homeostasis. Endocrine Rev (2013) 34(3):309–38. doi: 10.1210/er.2012-1055 PMC3660717 23460719

[23] Kulkoyluoglu-Cotul E Arca A Madak-Erdogan Z. Crosstalk Between Estrogen Signaling and Breast Cancer Metabolism. Trends Endocrinol Metab (2019) 30(1):25–38. doi: 10.1016/j.tem.2018.10.006 30471920

[24] Shieh A Epeldegui M Karlamangla AS Greendale GA. Gut Permeability, Inflammation, and Bone Density Across the Menopause Transition. JCI Insight (2020) 5(2):e134092. doi: 10.1172/jci.insight.134092 PMC7098720 31830000

[25] Zhao H Chen J Li X Sun Q Qin P Wang Q. Compositional and Functional Features of the Female Premenopausal and Postmenopausal Gut Microbiota. FEBS Lett (2019) 593(18):2655–64. doi: 10.1002/1873-3468.13527 31273779

[26] Diamanti-Kandarakis E. Hormone Replacement Therapy and Risk of Malignancy. Curr Opin Obstetrics Gynecol (2004) 16(1):73–8. doi: 10.1097/00001703-200402000-00013 15128011

[27] Vinogradova Y Coupland C Hippisley-Cox J. Use of Hormone Replacement Therapy and Risk of Breast Cancer: Nested Case-Control Studies Using the Qresearch and Cprd Databases. BMJ (2020) 371:m3873. doi: 10.1136/bmj.m3873 33115755 PMC7592147

[28] Flores R Shi J Fuhrman B Xu X Veenstra TD Gail MH. Fecal Microbial Determinants of Fecal and Systemic Estrogens and Estrogen Metabolites: A Cross-Sectional Study. J Trans Med (2012) 10:253. doi: 10.1186/1479-5876-10-253 PMC3552825 23259758

[29] Huang J Shan W Li F Wang Z Cheng J Lu F. Fecal Microbiota Transplantation Mitigates Vaginal Atrophy in Ovariectomized Mice. AGING-US (2021) 13(5):7589–607. doi: 10.18632/aging.202627 PMC7993734 33658399

[30] Wang Z Wang Q Zhao J Gong L Zhang Y Wang X. Altered Diversity and Composition of the Gut Microbiome in Patients With Cervical Cancer. AMB Express (2019) 9(1):40. doi: 10.1186/s13568-019-0763-z 30904962 PMC6431307

[31] Kang GU Jung DR Lee YH Jeon SY Han HS Chong GO. Dynamics of Fecal Microbiota With and Without Invasive Cervical Cancer and Its Application in Early Diagnosis. Cancers (Basel) (2020) 12(12):3800. doi: 10.3390/cancers12123800 PMC7766064 33339445

[32] Mirzaei R Afaghi A Babakhani S Sohrabi MR Hosseini-Fard SR Babolhavaeji K. Role of Microbiota-Derived Short-Chain Fatty Acids in Cancer Development and Prevention. BioMed Pharmacother (2021) 139:111619. doi: 10.1016/j.biopha.2021.111619 33906079

[33] Luu M Riester Z Baldrich A Reichardt N Yuille S Busetti A. Microbial Short-Chain Fatty Acids Modulate Cd8(+) T Cell Responses and Improve Adoptive Immunotherapy for Cancer. Nat Commun (2021) 12(1):4077. doi: 10.1038/s41467-021-24331-1 34210970 PMC8249424

[34] Park J Kim M Kang SG Jannasch AH Cooper B Patterson J. Short-Chain Fatty Acids Induce Both Effector and Regulatory T Cells by Suppression of Histone Deacetylases and Regulation of the Mtor-S6k Pathway. Mucosal Immunol (2015) 8(1):80–93. doi: 10.1038/mi.2014.44 24917457 PMC4263689

[35] Goff BA Mandel LS Melancon CH Muntz HG. Frequency of Symptoms of Ovarian Cancer in Women Presenting to Primary Care Clinics. Jama-Journal Am Med Assoc (2004) 291(22):2705–12. doi: 10.1001/jama.291.22.2705 15187051

[36] Xu S Liu Z Lv M Chen Y Liu Y. Intestinal Dysbiosis Promotes Epithelial-Mesenchymal Transition by Activating Tumor-Associated Macrophages in Ovarian Cancer. Pathog Dis (2019) 77(2):ftz019. doi: 10.1093/femspd/ftz019 30916767

[37] Gomes AC Hoffmann C Mota JF. The Human Gut Microbiota: Metabolism and Perspective in Obesity. Gut Microbes (2018) 9(4):308–25. doi: 10.1080/19490976.2018.1465157 PMC6219651 29667480

[38] Zahid M Beseler CL Hall JB LeVan T Cavalieri EL Rogan EG. Unbalanced Estrogen Metabolism in Ovarian Cancer. Int J Cancer (2014) 134(10):2414–23. doi: 10.1002/ijc.28565 PMC3949171 24170413

[39] Saeidi J Motaghipur R Sepehrian A Mohtashami M Forooghi Nia F Ghasemi A. Dietary Fats Promote Inflammation in Wistar Rats as Well as Induce Proliferation, Invasion of Skov3 Ovarian Cancer Cells. J Food Biochem (2020) 44(5):e13177. doi: 10.1111/jfbc.13177 32157714

[40] Chen L Zhai Y Wang Y Fearon ER Nunez G Inohara N. Altering the Microbiome Inhibits Tumorigenesis in a Mouse Model of Oviductal High-Grade Serous Carcinoma. Cancer Res (2021) 81(12):3309–18. doi: 10.1158/0008-5472.CAN-21-0106 PMC8260454 33863776

[41] Chambers LM Esakov EL Braley C Sangwan N Vargas R Rose PG. The Gut Microbiome Attenuates Epithelial Ovarian Cancer Growth and Platinum Sensitivity: Novel Opportunities for Ovarian Cancer Treatment. Gynecol Oncol (2020) 159:108–9. doi: 10.1016/j.ygyno.2020.05.113

[42] Tsunoda SM Gonzales C Jarmusch AK Momper JD Ma JD. Contribution of the Gut Microbiome to Drug Disposition, Pharmacokinetic and Pharmacodynamic Variability. Clin Pharmacokinet (2021) 60(8):971–84. doi: 10.1007/s40262-021-01032-y PMC8332605 33959897

[43] Zhao MF Liang GD Zhou YJ Chi ZP Zhuang H Zhu SL. Novel Bacillus Strains From the Human Gut Exert Anticancer Effects on a Broad Range of Malignancy Types. Invest New Drugs (2020) 38(5):1373–82. doi: 10.1007/s10637-020-00906-5 32734371

[44] Viaud S Saccheri F Mignot G Yamazaki T Daillere R Hannani D. The Intestinal Microbiota Modulates the Anticancer Immune Effects of Cyclophosphamide. Science (2013) 342(6161):971–6. doi: 10.1126/science.1240537 PMC4048947 24264990

[45] Raglan O Kalliala I Markozannes G Cividini S Gunter MJ Nautiyal J. Risk Factors for Endometrial Cancer: An Umbrella Review of the Literature. Int J Cancer (2019) 145(7):1719–30. doi: 10.1002/ijc.31961 30387875

[46] Tsilidis KK Kasimis JC Lopez DS Ntzani EE Ioannidis JP. Type 2 Diabetes and Cancer: Umbrella Review of Meta-Analyses of Observational Studies. BMJ (2015) 350:g7607. doi: 10.1136/bmj.g7607 25555821

[47] Yang X Wang J. The Role of Metabolic Syndrome in Endometrial Cancer: A Review. Front Oncol (2019) 9:744. doi: 10.3389/fonc.2019.00744 31440472 PMC6694738

[48] Lortet-Tieulent J Ferlay J Bray F Jemal A. International Patterns and Trends in Endometrial Cancer Incidence, 1978-2013. J Natl Cancer Inst (2018) 110(4):354–61. doi: 10.1093/jnci/djx214 29045681

[49] Plottel CS Blaser MJ. Microbiome and Malignancy. Cell Host Microbe (2011) 10(4):324–35. doi: 10.1016/j.chom.2011.10.003 PMC3264051 22018233

[50] Sarkar A Harty S Johnson KV Moeller AH Carmody RN Lehto SM. The Role of the Microbiome in the Neurobiology of Social Behaviour. Biol Rev Camb Philos Soc (2020) 95(5):1131–66. doi: 10.1111/brv.12603 PMC10040264 32383208

[51] Kisiela M Skarka A Ebert B Maser E. Hydroxysteroid Dehydrogenases (Hsds) in Bacteria: A Bioinformatic Perspective. J Steroid Biochem Mol Biol (2012) 129(1-2):31–46. doi: 10.1016/j.jsbmb.2011.08.002 21884790

[52] Fuhrman BJ Feigelson HS Flores R Gail MH Xu X Ravel J. Associations of the Fecal Microbiome With Urinary Estrogens and Estrogen Metabolites in Postmenopausal Women. J Clin Endocrinol Metab (2014) 99(12):4632–40. doi: 10.1210/jc.2014-2222 PMC4255131 25211668

[53] Shin JH Park YH Sim M Kim SA Joung H Shin DM. Serum Level of Sex Steroid Hormone Is Associated With Diversity and Profiles of Human Gut Microbiome. Res Microbiol (2019) 170(4-5):192–201. doi: 10.1016/j.resmic.2019.03.003 30940469

[54] Seyed Hameed AS Rawat PS Meng X Liu W. Biotransformation of Dietary Phytoestrogens by Gut Microbes: A Review on Bidirectional Interaction Between Phytoestrogen Metabolism and Gut Microbiota. Biotechnol Adv (2020) 43:107576. doi: 10.1016/j.biotechadv.2020.107576 32531317

[55] Chen KL Madak-Erdogan Z. Estrogen and Microbiota Crosstalk: Should We Pay Attention? Trends Endocrinol Metab (2016) 27(11):752–5. doi: 10.1016/j.tem.2016.08.001 27553057

[56] Bowey E Adlercreutz H Rowland I. Metabolism of Isoflavones and Lignans by the Gut Microflora: A Study in Germ-Free and Human Flora Associated Rats. Food Chem Toxicol (2003) 41(5):631–6. doi: 10.1016/s0278-6915(02)00324-1 12659715

[57] Senizza A Rocchetti G Mosele JI Patrone V Callegari ML Morelli L. Lignans and Gut Microbiota: An Interplay Revealing Potential Health Implications. Molecules (2020) 25(23):5709. doi: 10.3390/molecules25235709 PMC7731202 33287261

[58] Tapiero H Ba GN Tew KD. Estrogens and Environmental Estrogens. BioMed Pharmacother (2002) 56(1):36–44. doi: 10.1016/s0753-3322(01)00155-x 11905507

[59] Schreurs MPH de Vos van Steenwijk PJ Romano A Dieleman S Werner HMJ. How the Gut Microbiome Links to Menopause and Obesity, With Possible Implications for Endometrial Cancer Development. J Clin Med (2021) 10(13):2916. doi: 10.3390/jcm10132916 34209916 PMC8268108

[60] Nagashima M Miwa N Hirasawa H Katagiri Y Takamatsu K Morita M. Genome-Wide DNA Methylation Analysis in Obese Women Predicts an Epigenetic Signature for Future Endometrial Cancer. Sci Rep (2019) 9(1):6469. doi: 10.1038/s41598-019-42840-4 31015518 PMC6478742

[61] Modesitt SC Hallowell PT Slack-Davis JK Michalek RD Atkins KA Kelley SL. Women at Extreme Risk for Obesity-Related Carcinogenesis: Baseline Endometrial Pathology and Impact of Bariatric Surgery on Weight, Metabolic Profiles and Quality of Life. Gynecol Oncol (2015) 138(2):238–45. doi: 10.1016/j.ygyno.2015.05.015 26013696

[62] Laval L Martin R Natividad JN Chain F Miquel S de Maredsous CD. Lactobacillus Rhamnosuscncm I-3690 and the Commensal Bacteriumfaecalibacterium Prausnitziia2-165 Exhibit Similar Protective Effects to Induced Barrier Hyper-Permeability in Mice. Gut Microbes (2015) 6(1):1–9. doi: 10.4161/19490976.2014.990784 25517879 PMC4615674

[63] Martin R Chamignon C Mhedbi-Hajri N Chain F Derrien M Escribano-Vazquez U. The Potential Probiotic Lactobacillus Rhamnosus Cncm I-3690 Strain Protects the Intestinal Barrier by Stimulating Both Mucus Production and Cytoprotective Response. Sci Rep (2019) 9(1):5398. doi: 10.1038/s41598-019-41738-5 30931953 PMC6443702

[64] Spacova I Van Beeck W Seys S Devos F Vanoirbeek J Vanderleyden J. Lactobacillus Rhamnosus Probiotic Prevents Airway Function Deterioration and Promotes Gut Microbiome Resilience in a Murine Asthma Model. Gut Microbes (2020) 11(6):1729–44. doi: 10.1080/19490976.2020.1766345 PMC7524350 32522072

[65] Riaz Rajoka MS Zhao H Mehwish HM Li N Lu Y Lian Z. Anti-Tumor Potential of Cell Free Culture Supernatant of Lactobacillus Rhamnosus Strains Isolated From Human Breast Milk. Food Res Int (2019) 123:286–97. doi: 10.1016/j.foodres.2019.05.002 31284979

[66] Oh NS Joung JY Lee JY Kim Y. Probiotic and Anti-Inflammatory Potential of Lactobacillus Rhamnosus 4b15 and Lactobacillus Gasseri 4m13 Isolated From Infant Feces. PloS One (2018) 13(2):e0192021. doi: 10.1371/journal.pone.0192021 29444150 PMC5812581

[67] Dehghani N Tafvizi F Jafari P. Cell Cycle Arrest and Anti-Cancer Potential of Probiotic Lactobacillus Rhamnosus Against Ht-29 Cancer Cells. Bioimpacts (2021) 11(4):245–52. doi: 10.34172/bi.2021.32 PMC8494254 34631486

[68] Chenoll E Moreno I Sanchez M Garcia-Grau I Silva A Gonzalez-Monfort M. Selection of New Probiotics for Endometrial Health. Front Cell Infect Microbiol (2019) 9:114. doi: 10.3389/fcimb.2019.00114 31058101 PMC6481279

[69] Li N Pang B Li JJ Liu GW Xu XG Shao DY. Mechanisms for Lactobacillus Rhamnosus Treatment of Intestinal Infection by Drug-Resistant Escherichia Coli. Food Funct (2020) 11(5):4428–45. doi: 10.1039/d0fo00128g 32374342

[70] Hu MM Wu XL Luo M Wei H Xu D Xu F. Lactobacillus Rhamnosus Flrh93 Protects Against Intestinal Damage in Mice Induced by 5-Fluorouracil. J Dairy Sci (2020) 103(6):5003–18. doi: 10.3168/jds.2019-17836 32229117

[71] Urbancsek H Kazar T Mezes I Neumann K. Results of a Double-Blind, Randomized Study to Evaluate the Efficacy and Safety of Antibiophilus (R) in Patients With Radiation-Induced Diarrhoea. Eur J Gastroenterol Hepatol (2001) 13(4):391–6. doi: 10.1097/00042737-200104000-00015 11338068

[72] Cha MK Lee DK An HM Lee SW Shin SH Kwon JH. Antiviral Activity of Bifidobacterium Adolescentis Spm1005-A on Human Papillomavirus Type 16. BMC Med (2012) 10:72. doi: 10.1186/1741-7015-10-72 22788922 PMC3409845

[73] Nami Y Abdullah N Haghshenas B Radiah D Rosli R Khosroushahi AY. Assessment of Probiotic Potential and Anticancer Activity of Newly Isolated Vaginal Bacterium Lactobacillus Plantarum 5bl. Microbiol Immunol (2014) 58(9):492–502. doi: 10.1111/1348-0421.12175 25039934

[74] Sungur T Aslim B Karaaslan C Aktas B. Impact of Exopolysaccharides (Epss) of Lactobacillus Gasseri Strains Isolated From Human Vagina on Cervical Tumor Cells (Hela). Anaerobe (2017) 47:137–44. doi: 10.1016/j.anaerobe.2017.05.013 28554813

[75] Riaz Rajoka MS Zhao H Lu Y Lian Z Li N Hussain N. Anticancer Potential Against Cervix Cancer (Hela) Cell Line of Probiotic Lactobacillus Casei and Lactobacillus Paracasei Strains Isolated From Human Breast Milk. Food Funct (2018) 9(5):2705–15. doi: 10.1039/c8fo00547h 29762617

[76] Ankaiah D Esakkiraj P Perumal V Ayyanna R Venkatesan A. Probiotic Characterization of Enterococcus Faecium Por1: Cloning, Over Expression of Enterocin-A and Evaluation of Antibacterial, Anti-Cancer Properties. J Funct Foods (2017) 38:280–92. doi: 10.1016/j.jff.2017.09.034

[77] Brasseur K Auger P Asselin E Parent S Cote JC Sirois M. Parasporin-2 From a New Bacillus Thuringiensis 4r2 Strain Induces Caspases Activation and Apoptosis in Human Cancer Cells. PLoS One (2015) 10(8):e0135106. doi: 10.1371/journal.pone.0135106 26263002 PMC4532506

[78] Bermúdez-Humarán LG Langella P Cortes-Perez NG Gruss A Alcocer-Gonzales JM Tamez-Guerra RS. Immunisation Intranasale Chez La Souris Avec Des Lactocoques Exportant L’interleukine-12 Et L’antigène E7 Du Hpv-16. Le Lait (2004) 84(1-2):191–206. doi: 10.1051/lait:2003048

[79] Jang SE Jeong JJ Choi SY Kim H Han MJ Kim DH. Lactobacillus Rhamnosus Hn001 and Lactobacillus Acidophilus La-14 Attenuate Gardnerella Vaginalis-Infected Bacterial Vaginosis in Mice. Nutrients (2017) 9(6):531. doi: 10.3390/nu9060531 PMC5490510 28545241

[80] Lambert MNT Thybo CB Lykkeboe S Rasmussen LM Frette X Christensen LP. Combined Bioavailable Isoflavones and Probiotics Improve Bone Status and Estrogen Metabolism in Postmenopausal Osteopenic Women: A Randomized Controlled Trial. Am J Clin Nutr (2017) 106(3):909–20. doi: 10.3945/ajcn.117.153353 28768651

[81] Linn YH Thu KK Win NHH. Effect of Probiotics for the Prevention of Acute Radiation-Induced Diarrhoea Among Cervical Cancer Patients: A Randomized Double-Blind Placebo-Controlled Study. Probiotics Antimicrob Proteins (2019) 11(2):638–47. doi: 10.1007/s12602-018-9408-9 29550911

[82] Chitapanarux I Chitapanarux T Traisathit P Kudumpee S Tharavichitkul E Lorvidhaya V. Randomized Controlled Trial of Live Lactobacillus Acidophilus Plus Bifidobacterium Bifidum in Prophylaxis of Diarrhea During Radiotherapy in Cervical Cancer Patients. Radiat Oncol (2010) 5:31. doi: 10.1186/1748-717X-5-31 20444243 PMC2874795

